# Supplementary figures and images for: Identification of Clinical Variants beyond the Exome in Inborn Errors of Metabolism
Source: Int J Mol Sci. 2022 Oct 25;23(21):12850. doi: 10.3390/ijms232112850 (PMC9654865; doi:10.3390/ijms232112850)

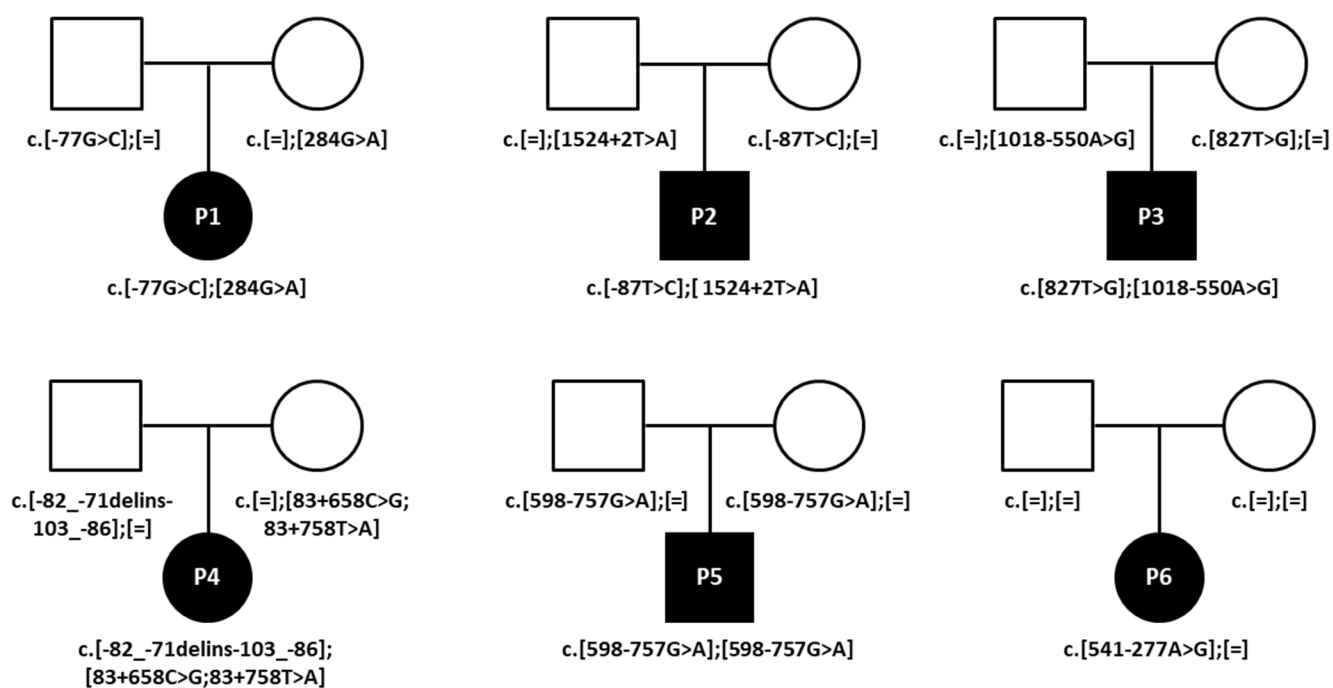

**Figure S1. Mendelian segregation of the six patients**

Supplement: Supplementary file 1 [file ijms-23-12850-s001.zip › Soriano-Sexto et al. Suplementary Figures.pdf]
